# Psychosocial wellbeing and physical health among Tamil schoolchildren in northern Sri Lanka

**DOI:** 10.1186/s13031-016-0081-x

**Published:** 2016-07-06

**Authors:** Alexander Hamilton, Charlie Foster, Justin Richards, Rajendra Surenthirakumaran

**Affiliations:** British Heart Foundation Centre of Population Approaches to NCD Prevention, University of Oxford, Oxford, UK; School of Public Health and Charles Perkins Centre, University of Sydney, Sydney, Australia; Department of Community and Family Medicine, Faculty of Medicine, University of Jaffna, Jaffna, Sri Lanka

**Keywords:** Sri Lanka, Mental health, Conflict, Physical health

## Abstract

**Background:**

Mental disorders contribute to the global disease burden and have an increased prevalence among children in emergency settings. Good physical health is crucial for mental well-being, although physical health is multifactorial and the nature of this relationship is not fully understood. Using Sri Lanka as a case study, we assessed the baseline levels of, and the association between, mental health and physical health in Tamil school children.

**Methods:**

We conducted a cross sectional study of mental and physical health in 10 schools in Kilinochchi town in northern Sri Lanka. All Grade 8 children attending selected schools were eligible to participate in the study. Mental health was assessed using the Sri Lankan Index for Psychosocial Stress – Child Version. Physical health was assessed using Body Mass Index for age, height for age Z scores and the Multi-stage Fitness Test. Association between physical and mental health variables was assessed using scatterplots and correlation was assessed using Pearson’s R.

**Results:**

There were 461 participants included in the study. Girls significantly outperformed boys in the MH testing *t* (459) = 2.201, *p* < 0.05. Boys had significantly lower average Body Mass Index for age and height for age Z scores than girls (BMI: *t* (459) = −4.74, *p* <0.001; Height: *t* (459) = −3.54, *p* < 0.001). When compared to global averages, both sexes underperformed in the Multi-Stage Fitness Test, and had a higher prevalence of thinness and stunting. We identified no meaningful association between the selected variables.

**Conclusions:**

Our results do not support the supposition that the selected elements of physical health are related to mental health in post-conflict Sri Lanka. However, we identified a considerable physical health deficit in Tamil school children.

## Background

Mental health disorders are a crucial contributor to disability and mortality, as well as to accidental and non-accidental injuries globally [1]. Armed conflict and the ensuing conditions are risk factors for poor mental and physical health in adolescents and children [2–5]; in fact, the prevalence of mental disorder tends to double in emergency and post-conflict settings [6]. Conflict not only exposes adolescents to violent and traumatic events which are known to increase mental health problems, but can also radically change the social and economic environment which can catalyse the emergence of psychiatric disorders [7, 8].

Recent cross sectional research indicates that the causal relationship between trauma and child and adolescent mental health is not simple; family functioning and other adversities may also independently impair mental health. Previous studies in Sri Lanka have identified high levels of exposure to war-related events and concurrent mental disorder in children [9, 10]. In addition, several studies support the notion that multiple traumatic exposures compound to produce a higher likelihood of mental disorder [11, 12]. Importantly, experience of traumatic events and mental health disorders are associated with cognitive impairment and school absenteeism [13]. Studies suggest, however, that everyday stressors may partially mediate the relationship between war-related exposures and mental health outcomes, and that exposure to mass trauma and experiences of family violence are independently associated with mental health problems in children [14–16].

The inclusion of secondary adversities as moderators in the trauma exposure and mental health relationship may mistakenly downplay the importance of risk and protective factors. It is not possible to infer levels of mental health and well-being based on the presence or absence of risk and protective factors alone; two people exposed to identical environmental conditions can display different mental health profiles [17]. Mental health is determined by a broad range of social, economic, and environmental factors on an individual, community and social level; these factors work in manifold interacting processes [18]. Identifying specific, changeable risk and protective factors may be crucial in addressing mental health problems in children.

According to the UN Convention on the Rights of the Child [19], every child has the right to the best possible health and opportunity for developing to their full potential, especially those affected by armed conflict. Good physical health has been identified as a crucial factor for promoting mental health in LMIC contexts [18, 20]. Physical health is a measure composed of multiple, interacting components and its relationship with mental health is complex and multifaceted [21]. Many physical health conditions increase the risk for mental disorder, whilst mental health conditions complicate health seeking behaviours, lifestyle behaviours and treatment outcomes [21, 22]. In this particular study, we selected two indices of physical health that can be reliably assessed in children in a post-conflict context, malnutrition and physical fitness.

High prevalence of malnutrition is common in conflict and post-conflict situations [23]. Socio-economic status and living conditions are strongly correlated with mental health in young people [3, 24]. Among children and adolescents, malnutrition, particularly thinness, is a feasible proxy indicator of a difficult home life that lacks sufficient available food. We identified no studies that assessed the association between adolescent malnutrition and mental health in post-conflict states, despite its crucial role in adolescent psychological development [25]. In the absence of evidence regarding malnutrition, research indicates that food insecurity is associated with poor mental health outcomes; it is a predictor of depression, suicidal ideation and low mood in adolescents [26].

Physical activity impacts mental health through a variety of mechanisms including psychological, psychosocial, pharmacological and physiological [27]. Physical fitness is the physiological outcome of increased physical activity [28]. The promotion of physical activity and fitness may improve mental health in several ways: through the promotion of self-esteem, increased resilience, improved self-worth and image, and it has a protective effect against mental disorder, especially depressive symptoms [29, 30]. Meta-analytic evidence from HICs suggests a positive impact of physical activity interventions on self-esteem in young people [31]. RCT evidence indicates that physical activity significantly reduces depression, anxiety and can increase self-esteem in young people [32]. Cross-sectional evidence supports the relationship between fitness, activity and depression in HICs; a longitudinal study in adolescents identified a protective effect of cardiorespiratory fitness against depression [33]. There is also emerging evidence of an association between physical fitness and mental health in LMICs [34].

Although there is now a growing body of evidence for the various positive impacts of physical activity on the physical, mental and social wellbeing of young people, including those ‘at-risk’ in developed, peaceful settings [30, 31, 35–38]; the relationship between physical activity and mental health in post-conflict settings remains unclear [39, 40]. It is not clear whether the associations identified in HIC settings are externally valid and relevant for adolescents living in post-conflict conditions.

Increasingly, physical activity and exercise-based interventions are incorporated into assistance for conflict-affected populations; the evidence base for these interventions is scant [41]. Several publications support the notion that physical activity can act as a buffer in the relationship between stressors and mental illness [42, 43].

In the post-conflict context, the promotion of fitness through physical activity and sport might represent a potential intervention lever, it may alleviate stress and assist with reducing the symptoms of mental disorder [44]. It is essential to test these assumptions prior to the use of physical activity to promote positive mental health among young people in post-conflict contexts. Coordinating an appropriate response to the challenges that mental disorders present requires an understanding of risk and protective factors for poor mental health [45].

In this study we assessed psychological distress and physical health in Grade 8 students in Killinochchi, using a locally validated measure of mental health. Secondary aims were to explore the associations between both physical fitness and malnutrition and mental health. In 2009, Sri Lanka emerged from a prolonged civil conflict between Government forces and the Liberation Tigers of Tamil Eelam (LTTE). During the conflict thousands of people died, were injured, or went missing; many more were displaced, often multiple times [46]. Previous research in adult and adolescent populations has identified variable levels of common and severe mental disorders dependent, in part, on exposure to traumatic events and current life situation [46–54].

We predict a high prevalence of stunting and malnutrition among young adolescents in Kilinochchi as a result of the high exposure to adverse conditions. Previous studies of BMI in Sri Lanka have identified geographic variation. “Jayatissa and Randbanda [55] conducted a large scale, representative study of BMI for age and height for age in Sri Lanka. Prevalence of underweight (defined in the study as <5th percentile of BMI) in 13 year-old boys and girls was 66.5 and 26.4 % respectively; prevalence of stunting (<3rd percentile) was 41.3 and 40.1 % [55]. Further, a cross-sectional study in Mullaitivu identified a large proportion of underweight (-2 standard deviations (SD) weight-for-age in this study), 34 %, in 6-18 year olds [56].”

We predict that young adolescents who are malnourished will have worse mental health than healthy weight adolescents in the target population. Food insecurity and malnutrition are positively correlated in Sri Lanka [57]. Previous research in among displaced populations in Sri Lanka identified an association between food insecurity and common mental health disorders in adults, notably depression, and this association persisted in return migrants one year later [49, 50]. Wickramage et al. [58] assessed the prevalence of malnutrition and mental disorder in children left behind by international labour migrants. Children from migrant families exhibited significantly higher prevalence of emotional problems and hyperactivity disorders than comparative families, although the study did not assess the relationship between malnutrition and mental health. We identified no studies that assess the relationship between malnutrition and poor mental health in young adolescents, despite evidence from Sri Lanka and developed settings suggesting the two may well be associated.

A review of physical activity prevalence in South Asian children and adolescents identified regional decreases in average activity, although only one publication from Sri Lanka was included [59, 60]. The study relied on parent-reported data and identified ‘satisfactory levels’ of physical activity in children aged 8–12 in Colombo. We identified no studies of fitness in Sri Lankan children and adolescents. Further, we identified no studies that assess the relationship between mental health and physical activity, or fitness, in Sri Lanka. In the absence of fitness or activity data we cannot comment on the potential of physical activity for mental health promotion in this context. Based on regional data we predict a low level of fitness in the target group, due to decreasing levels of physical activity in the South Asian region. Further, we predict an association between physical fitness and mental health in the target population, as current research indicates a positive association between physical activity and positive aspects of mental health.

## Methods

We aimed to collect valid and reliable data within the practical and logistical constraints of the local context in Kilinochchi. This involved capturing information quickly, and for a large number of participants, with minimal disruption to school time and reliance on electrical equipment. Kilinochchi town was selected because it was the site of the LTTE headquarters during the civil war and capital of the de facto Tamil state for many years. As a result, it experienced some severe fighting and at one point the majority of the population was displaced [49]. After consultation with local stakeholders, we selected schools as the data collection site; participation rates are high (91.4 % for ages 10–14) and this maximized reach in a changeable context [61].

We stratified according to school type. State schools in Sri Lanka are divided into three streams based on the subjects they cover and the age span of the students they teach. There are three main types of school: AB, C and 2. Type AB schools are considered the best-equipped schools and teach from Grade 1 to Grade 13 (Advanced Level). Type C schools also teach to Advanced Level, but only Arts subjects. Type 2 schools teach all subjects to Ordinary Level, from grades 1–11 [62]. The ratio of Type AB, C and 2 schools was 1:1:2 in Kilinochchi town. We chose this sampling method to ensure all the relevant school types were included in the study.

Ten schools were randomly selected from the Kilinochchi town schools register using random number tables. The register was obtained from the Office of Education for Kilinochchi. The decision to collect data in 10 schools was based on several factors. The Principal Investigator (PI) for the study was only permitted access to Kilinochchi for a short period of time, due to restrictions on international researchers during the data collection period. The ethics process, feasibility testing and data collection team training used up a considerable portion of this allotted time. The knock on effect was that there were only two weeks available for data collection prior to the beginning of the school holidays, after which data collection would no longer be possible. It was a practical decision based on the time available to the researchers, and the capacity of the research team to collect data in one school per day.

Grade 8 students were selected as, at the time of the data collection, a local development organisation was planning sports-based programmes for this specific target group. The cross-sectional study was doubling up as baseline measurements for the organisation. All Grade 8 students in included schools were eligible for participation, unless we were informed otherwise by their teacher or guardian.

### Participants

Students were recruited from 1 to 7^th^ of October 2014 and data collection took place from the 6^rd^ – 17^th^ of November 2014. All grade 8 students enrolled in a selected government schools were eligible for inclusion in the study.

### Outcome variables

Mental health was assessed using a locally designed and validated psychosocial assessment tool, the Sri Lankan Index of Psychosocial Status – Child version (SLIPS-C) [16, 53]. The SLIPS-C is a 49-item Likert scale self-complete questionnaire in which participants rate the frequency of feelings, behaviours and emotions over the preceding two weeks on a scale from 0 to 4 (never-almost always). The internal consistency of the scale is high (Cronbach’s alpha – Tamil Version = 0.85). The SLIPS-C is comprised of three subscales: internalising, externalising and withdrawal behaviour. In this study, we only reported the total SLIPS-C score and externalising subscale score. Previous research in other cohorts has identified higher levels of externalising behaviours (EXT) in young boys than young girls [63].

Assessment of basic demographic data such as age, sex, ethnicity and religious group is attached to the SLIPS-C. The SLIPS-C was selected in co-ordination with the local NGO planning a sports programme. The NGO felt it reflected the aims of their intended intervention well, and would provide locally relevant information.

Physical health measures included body composition and cardiovascular fitness. Body composition was approximated using Body Mass Index (BMI) for age Z score (BFA) and height for age Z score (HFA). A BMI Z score indicates how many units of the standard deviation an individual BMI score is above or below the average BMI for their age group, which are derived from a global population. We used the WHO global standard growth references and cut offs for stunting and wasting [64]. We felt the WHO cut offs were the most appropriate for this study, as a large sample of Indian children and adolescents were included in the formation of the global averages. HFA is an indicator of chronic nutrition status, while BFA is an indicator of current nutritional status (malnutrition in this case). Comparing the proportions of our sample in different HFA and BFA categories with global and regional populations gives an estimate of nutritional status in the area [64].

Calculation of BFA and HFA require the measurement of height, weight and age. The protocols for the measurement of height and weight have been standardized, are easily reproducible and require minimal training for delivery [65]. Cardiovascular fitness was measured using the Multi-stage fitness test (MFT) [66]. MFT global average scores are stratified by sex and age, as there are gendered and age-related differences in performance. The standardized MFT has been found to be a reliable and valid method to assess cardiovascular fitness in school-age populations [67, 68].

### Study procedure

The Department of Community and Family Medicine, University of Jaffna provided logistical support, including 5^th^ year Medical students for data collection. All team members received two days theoretical training and three days practical training at a local school. A small repeat measures study (*n* = 30) conducted at a convenient school indicated the reliability of the measurement methods. Inter-tester agreement was assessed using the Inter-Class Correlation Coefficient. The level of agreement for physical assessment methods was excellent or very good (*r* =0.993 for weight and height, *r* = 0.78 for the MFT) and good for mental health (*r* = 0.701).

On the day of testing, a research team leader briefed all participants. Participant assent was taken prior to any measurement. Students were given a test sheet to complete, including demographic information and the SLIPS-C, and guided through completion of the form. Physical testing began once all students had completed the SLIPS-C. The PI and a data collection team member checked that all items on the SLIPS-C were answered before student proceeded to physical health testing. This was a measure to avoid missing data, which had been identified as a problem during feasibility testing. If any items were left unanswered, participants were encouraged to complete the item away from their peers to avoid pressure.

### Ethics approval

Prior to contacting any school all the relevant permissions were sought from local authorities. The PI visited the school principals prior to the testing date to obtain permission and to explain the purpose of the study. Class teachers were given information sheets and consent forms in the relevant local language. Participants were also given information sheets at this time. Parental or guardian informed consent was required for participation in the study.

Participant data were encoded and stored on an encrypted laptop. Completed surveys and identifiers were stored separate, locked cabinets. Data was accessible only by the lead researcher, except during the double entry process. The University of Oxford Tropical Research Ethics Committee and the University of Colombo Ethics Review Committee granted ethical approval for the study. Ref no: EC-14-074.

### Statistical methods

The Statistical Package for the Social Sciences 7 (SPSS) was used to enter and analyse data [69]. Descriptive statistics (mean, SD and 95 % confidence intervals) were calculated for all outcome measures. The use of ANOVA and the independent *t*-test requires that the data are normally distributed and the samples under comparison have homogeneity of variance. Normality and homogeneity assumptions were verified prior to commencing analysis, using the Kolmogorov-Smirnoff and Levene’s tests respectively.

SLIPS-C, BFA, HFA and MFT scores were stratified by sex. Internal validity of the SLIPS-C was assessed using Cronbach’s Alpha. The mean and 95 % confidence intervals were calculated and compared for boys and girls, as a previous cross sectional study with the SLIPS-C has identified significant gender differences [16]. Data for comparisons between Ampara district and Kilinochchi were extracted from the previous publication using the SLIPS-C. An independent *t*-test was used to identify differences in mental and physical health performance in boy and girls. BFA and HFA Z scores were calculated using STATA, and proportions in each thinness and stunting category were compared to provide an overview of acute and chronic nutritional status. Global comparisons of BFA and HFA only used 13-year-old Grade 8 students, as the global average scores are stratified by age.

Association between physical health variables and mental health was initially assessed using scatterplots. Correlation was statistically assessed using Pearson’s r. Mean mental health scores of ‘malnourished’ (BFA < = − 2SD) and ‘normal weight’ (BFA > −2SD), and ‘severely malnourished’ (BFA < = − 3 SD) and ‘normal weight’ groups were compared using ANOVA. We compared mean mental health scores of ‘fit’ (MFT score > −1SD of global average score) and ‘unfit’ (MFT score < −1 SD of global average) boys and girls.

The SLIPS-C has specific data cleaning rules designed to ensure the participant properly comprehends the study and is focusing. The rules were outlined by Dr Gaithri Fernando, the author of the SLIPS-C, in personal communication. There are two items that are designed to ensure the participant properly comprehends the questions: ‘I saw birds’ and ‘I told lies’. If a student answers either of these items with a 0 (e.g., ‘I did not see birds at all over the last two weeks’) then the questionnaire is dropped. In addition, there is a repeat item ‘I played with my friends in the last two weeks’. If there is more than a 2-point discrepancy between the two item scores, the questionnaire is dropped on the basis that the individual was not paying attention.

In the event that an individual was missing either one or two items in the SLIPS-C, a score of 0 was imputed. In the event that there were three or more answers missing, the questionnaire was not included in the final analyses as the feasibility testing indicated that if participants made a few mistakes on the answer sheet, they were likely to lose their place and answer the questions incorrectly.

## Results

Six hundred eighty-three students were eligible for inclusion in the study. 537 (78.6 %) were present on the day of testing and gave consent. After cleaning and removal of outliers, 461 cases remained. There were only 3 cases in which the participant had incompletely filled out the questionnaire after checking. Sociodemographic information is tabulated in Table 1. Mean values for all outcome variables are reported in Table 2.

**Table 1:** Sample sociodemographic information

Socio-demographic variables	Number	% of sample
Gender		
Male	227	(49.2)
Female	234	(50.7)
Religion		
Hindu	337	(72.4 %)
Christian	125	(27.1 %)
School type		
1 AB	156	(33.8 %)
1C	84	(18.2 %)
2	221	(47.9 %)

**Table 2:** Mean scores and 95 % confidence intervals for outcome variables in Kilinochchi (*n* = 461)

Outcome	Boys	Girls
n	227	234
Mental health scores		
MH	61.28*	56.73
	58.64–64.22	53.91–59.55
EXT	12.73	11.58
	11.76–13.69	10.75–12.37
Physical health scores		
MFT score	10.03*	9.08
	9.92–10.14	8.93–9.23
BMI	16.32*	17.53
	16. 05–16.59	17.2–17.86
BFA	−1.43*	−0.9
	- 1.59–1.27	- 1.05–0.75
HFA	−1.54*	−1.21
	−1.82–1.26	−1.33–1.09

Bold indicates a significant difference between boys and girls scores (* = *p* < 0.05)

### Mental health scores

Girls significantly outperformed boys in the MH testing in the target group *t* (459) = 2.201, *p* < 0.05 and the SLIPS-C demonstrated a high level of internal consistency in this population and context (α = 0.868). There was no significant difference in externalising behaviour scores between boys and girls *t* (459) = 3.17, *p* = 0.76.

### Physical health scores

Boys significantly outperformed girls in the MFT (*t* (459) = 9.84, *p* < 0.01). Boys had significantly lower average BHA and HFA Z scores than girls (BFA: *t* (459) = −4.74, *p* <0.001; HFA: *t* (459) = −3.54, *p* < 0.001). When compared to global averages, both sexes significantly underperformed in the MFT. Grade 8 BFA Z scores were on average −1.43 (+/− 0.16) SD below the global normative values. Similarly, Grade 8 girls BFA Z score was −0.90 (+/− 0.15) SDs below the global norm. HFA scores in both sexes were poor (boys: −1.54 +/− 0.14; girls −1.21 +/− 0.12). Table 3 indicates the very high levels of stunting and thinness among both boys and girls when compared to global averages. Further, girls had significantly higher BMI scores than boys (*t* (459) = −5.523, *p* < 0.01).

**Table 3:** Proportion of sample in each BFA and HFA category compared to global normative values (*n* = 434)

WHO category	Proportions in each BFA for age category		
	Boys aged 13 (%)	Girls aged 13 (%)	Global normative value (%)
Severely thin	10	4	0.14
Thin	31.8	15.2	2.14
Normal weight	56.3	75	81.86
Overweight	1.9	5.4	13.59
Obese	0	0	2.28
	Proportions in each HFA category		
Severely stunted	3.8	3.1	0.14
Stunted	36.5	17.9	2.14
Normal height	59.7	79	95.45
2SD < HFA < 3SD	0	0	0.14
Severely stunted	3.8	3.1	0.14

This table indicates the proportions of the Kilinochchi sample (*n* = 461) in each of the BFA and HFA categories according to the WHO global normative values, stratified by age

### Association between mental and physical health variables

Initial assessment with scatter plots indicated that there was no significant association between either cardiorespiratory fitness or BFA and mental health score. Pearson’s R indicated that MFT score did not explain a significant amount of variation in mental health score (*r* = −0.019, *p* = 0.691), but BFA did (*r* = −0.098, *p* = 0.035). BFA predicted such a small amount of mental health variation that we did not conduct multiple regression analyses as the relationship was practically insignificant.

### Comparison of groups stratified by physical health

Table 4 outlines SLIPS-C scores stratified by nutrition and fitness status. ANOVA confirmed that malnourished children have worse SLIPS-C scores than ‘healthy weight’ children (F 3.97, *p* = 0.047). Severely malnourished children (−3SD) performed significantly worse than healthy weight children (*F* =6.72, *p* = 0.01). However, when the results were stratified by gender, the differences between the malnourished group and the healthy group are no longer significant. Table 5 outlines SLIPS-C score, stratified by gender and fitness level. There was no significant difference in the SLIPS-C scores of different fitness groups, stratified by gender.

**Table 4:** SLIPS-C scores of healthy weight and malnourished participants

	Number	SLIPS-C score
Healthy weight participants	351	57.82
		55.57–60.07
Malnourished participants	110	***62.65***
		58.02–67.29
Severely malnourished	34	***68.03***
		58.73–77.33

This table indicates the SLIPS-C scores of the Kilinochchi sample stratified by nutrition status. NB ‘malnourished’ analysis includes all participants that were also severely malnourished. Bold and italics indicates a significant difference between value and ‘healthy weight’ values to the 0.05 significance level

**Table 5:** SLIPS-C scores of fit and unfit participants

		Fit	Unfit
SLIPS-C score	Girls	57.37	56.17
		53.55–61.19	52.02–60.32
	Boys	59.21	63.09
		55.09–63.32	58.90–67.30

This table indicates the SLIPS-C scores of the Kilinochchi sample stratified by fitness status

## Discussion

The present study investigated the levels of, and association between, mental and physical health parameters in young adolescent Tamils in Kilinochchi town. Our main finding was that there was a significant physical health deficit in the target population, although neither malnutrition nor cardiorespiratory fitness was associated with mental health in a meaningful manner. The present findings reaffirm the harmful consequences of conflict on the nutritional status of young adolescents, and suggest that adolescents in Kilinochchi town are not engaging in sufficient health-promoting physical activity.

A previous study using the SLIPS-C assessed young adolescents in Ampara, Eastern Sri Lanka [16]. There was no difference in the mean SLIPS-C scores between young adolescents in Kilinochchi and Ampara (Kilinochchi mean: 58.97 +/− 2.04; Ampara mean: 50.83 +/− 12.4). Ampara was also affected by the conflict, and the 2004 Tsunami, and so it is perhaps unsurprising that there are not significant differences in the mental health profile of young people in these regions. In accordance with the previous study using the SLIPS-C, girls outperformed boys in mental health testing [16]. Previous research has identified gendered differences in mental health and distress in conflict-affected and non-conflict-affected adolescents [58]. Depressive symptoms are more prevalent among adolescent girls, whereas boys tend to be more susceptible to high risk behaviours and suicide [70, 71]. In younger children, boys are more susceptible to hyperactivity disorders and externalising behaviour, although our results did not support this inference as there was no significant difference in the externalising subscale scores of boys and girls.

The selection of an emic measure of mental health is a double-edged sword; it may paint a locally relevant picture of mental health, but obstructs international comparisons. The selection of such a measure limits the external validity of the findings of the study. Equally, there are few studies using SLIPS-C, which limits the amount of comparable data available. The SLIPS-C has only been used a cross sectional measure before, and cut off points for clinically significant levels of distress have not yet been identified. As a result we can provide little comment on the prevalence of mental disorder in the target population with reference to other populations of young adolescents, only differences within our target group. This is the principal weakness in the conception of this study, but was a necessary concession to satisfy local partners. The SLIPS-C was initially selected as a metric for use by a local NGO as an indicator of programme impact, rather than a screening tool for high risk individuals.

Before any further work with the SLIPS-C is commenced, it should be validated with previously validated tools, such as the strengths and difficulties questionnaire (SDQ), which has been used successfully elsewhere in Northern Province and other parts of Sri Lanka [11, 14, 72, 73]. In this study, we did not use the SDQ as child self-reported scores require triangulation with parent and teacher reporting. This is a strength of the SDQ, although our local partners did not feel it was feasible to use this measure in the time frame allotted for the study.

In line with previous studies of malnutrition, children in conflict-affected Kilinochchi performed poorly when compared to global and regional normative scores; 41.8 % of boys and 19.2 % of girls were thin or severely thin when compared to a global average value of 2.28 % [74]. In this study, boys performed worse than their regional contemporaries in Mullaitivu, although comparisons with the general Sri Lankan population are complicated as different methods of assessing underweight were employed in nationally representative study. [56]. Previous research in South Asia has identified higher levels of thinness and stunting in girls than boys [75, 76]. Our data are not consistent with this trend, although support the results of previous research in Sri Lanka in which more girls are of normal BFA than boys [55, 77].

Higher rates of thinness in Kilinochchi may be the result of incomplete coverage of food programmes in northern Sri Lanka, and food insecurity continues to be an important driver of malnutrition in children [78]. Malnutrition continues to be rife within young people in Kilinochchi, despite increased efforts at providing support for struggling families and other high risk groups [57].

Contrary to our prediction, this study did not find an association between mental health and malnutrition in Grade 8 students in Kilinochchi. This may be because malnutrition is not a determinant of mental health, or that the selected metrics fail to capture the relationship appropriately. Comparison of crude mean mental health scores between malnourished (<−2SD) and healthy weight (> − 2SD) children suggested that malnourished children have a lower average mental health score; although grouping the results into weight categories may have affected the results. Specifically, grouping the data means that the relationships will be more affected by outliers, artificially enhancing mean differences between groups. When all the participants are considered simultaneously, as in a Pearson’s R analysis, there is no meaningful relationship between malnutrition and mental health.

The results suggest that BFA is not a predictor of mental health in the target population. Further research utilising other measures of mental health may provide further insight into this potential association. Results from HICs indicate that food insecurity and mental disorders are related in adolescents, although malnutrition may not be the explanatory factor in this relationship. In fact, it may be other elements of the home and socio-economic environment associated with food insecurity that drive its association with mental health in other contexts.

As predicted, both girls and boys scored poorly on the MFT, indicating low levels of cardiorespiratory fitness. The findings of the current study indicate that young adolescents Tamils in northern Sri Lanka are not participating in sufficient health promoting physical activity. This may be the result of many factors, and possibly due to the low priority of physical activity in Sri Lankan schools.

Cardiorespiratory fitness was not associated with mental health and this suggests it may not be an important protective factor in this specific population. Identification and dissemination of non-significant associations is crucial to prevent the proliferation of publication bias, and to avoid research duplication [79]. A recent systematic review identified 3 publications that detail the use of physical activity and sport to promote mental health in post conflict countries [41]. The studies were of variable quality and had equivocal results. Physical activity is increasingly incorporated into assistance for conflict-affected adolescents without a clear understanding of the relationship between activity and mental health. The results of this study do not support the use of physical activity alone to protect against psychosocial stress. However, given the level of evidence in this study, we cannot draw firm conclusions about this association and the role of physical activity remains unclear.

### Limitations

The research context introduced many broad methodological challenges, which impacted upon study design and data collection. Due to logistical and political constraints, it was not possible to sample wider than Kilinochchi town. As a result, our data are not representative of the whole community of Sri Lankan Tamil adolescents. Further, we were only able to sample in Grade 8 students in Kilinochchi, and as a result unable to identify any significant age-related relationships. The limited access to research sites as a result of political restrictions detracted from the quality of the sampling and the design of the study. Our study had a high level of absenteeism, which may have influenced the representativeness of the sample and underestimated the prevalence of mental disorder, as previous research indicates that school absenteeism is positively associated with worse mental health status [13].

There were significant limitations to the measures chosen in this study. The MFT is a reliable measure of cardiorespiratory fitness, but is only a proxy measure of physical activity. Physical activity may have a relationship with mental health that is independent of fitness. However, the use of the MFT was warranted in this study for practical reasons, and fitness is also a more permanent characteristic, whereas activity is highly variable day to day.

In this study, the MFT was often completed in sub-optimal conditions; children were often bare foot and, at times, running on loose sand or uneven ground. The feasibility testing indicated that the MFT is a reliable measure in this context, but may be subject to systematic error. The MFT global average is devised from multiple studies, largely in developed settings in which the test is conducted in sports halls with appropriate footwear. Participants in Kilinochchi may consistently underperform as a result of test conditions, rather than as a result of inferior fitness [80].

The measurement of height and weight was shown to be highly reliable and reproducible in the formative testing for this study. However, the WHO BFA and HFA cut offs may be inappropriate for this sample, as they may overestimate the level of thinness and stunting [81]. The cut offs may be doubly inappropriate because the target population is both South Asian and living in post-conflict setting. In our opinion, the results indicate high rates of malnutrition and stunting in Kilinochchi regardless of the limitations of the WHO cut offs, which we felt were the most appropriate available.

The data cleaning method outlined by Fernando is particularly stringent and excludes large numbers of participants from analyses; 69 participants were screened out because they failed to respond appropriately to the repeat item in the SLIPS-C. With such stringent cleaning criteria, there is a chance that children suffering from worse levels of mental health are excluded from analyses.

## Conclusions

Our results do not support the supposition that certain elements of physical fitness are related to mental health in post-conflict Sri Lanka. However, our results must be read with caution, given that they are representative of only a very specific population. Despite the lack of meaningful interdependence between the outcome variables, we identified important independent deficits in the target population.

Despite the non-significant findings, it is hoped that these preliminary results will indicate a need for further research into physical activity and mental health in northern Sri Lanka. This research indicates that fitness is not associated to psychosocial well-being in this specific population. However, the relationship between physical activity and specific mental disorders warrants exploration.

Likewise, as Sri Lanka continues to develop and urbanise, young people are at a heightened risk of physical inactivity, mental health issues and associated non-communicable disease risk factors [82]. There is a palpable lack of physical activity research in Sri Lankan adolescents and they are assumed to be sufficiently physically active. The present physical health findings require replication in larger and more representative samples to properly identify the distribution of poor physical fitness. Future research in this area should aim to fill this epidemiological gap to identify future health promotion priorities in Sri Lanka.
